# Design Principles in mHealth Interventions for Sustainable Health Behavior Changes: Protocol for a Systematic Review

**DOI:** 10.2196/39093

**Published:** 2023-02-22

**Authors:** Lin Yang, Angela Kuang, Claire Xu, Brittany Shewchuk, Shaminder Singh, Hude Quan, Yong Zeng

**Affiliations:** 1 Department of Cancer Epidemiology and Prevention Research Alberta Health Services Calgary, AB Canada; 2 Department of Oncology Cumming School of Medicine University of Calgary Calgary, AB Canada; 3 Department of Community Health Sciences Cumming School of Medicine University of Calgary Calgary, AB Canada; 4 Centre for Health Informatics, Cumming School of Medicine University of Calgary Calgary, AB Canada; 5 School of Nursing and Midwifery Faculty of Health, Community and Education Mount Royal University Calgary, AB Canada; 6 Department of Community Health Science Cumming School of Medicine University of Calgary Calgary, AB Canada; 7 Concordia Institute for Information Systems Engineering Concordia University Montreal, QC Canada

**Keywords:** behavior change, intervention, mHealth, personalization, dialogue, mobile health, mobile app, self-management

## Abstract

**Background:**

In recent years, mHealth has increasingly been used to deliver behavioral interventions for disease prevention and self-management. Computing power in mHealth tools can provide unique functions beyond conventional interventions in provisioning personalized behavior change recommendations and delivering them in real time, supported by dialogue systems. However, design principles to incorporate these features in mHealth interventions have not been systematically evaluated.

**Objective:**

The goal of this review is to identify best practices for the design of mHealth interventions targeting diet, physical activity, and sedentary behavior. We aim to identify and summarize the design characteristics of current mHealth tools with a focus on the following features: (1) personalization, (2) real-time functions, and (3) deliverable resources.

**Methods:**

We will conduct a systematic search of electronic databases, including MEDLINE, CINAHL, Embase, PsycINFO, and Web of Science for studies published since 2010. First, we will use keywords that combine mHealth, interventions, chronic disease prevention, and self-management. Second, we will use keywords that cover diet, physical activity, and sedentary behavior. Literature found in the first and second steps will be combined. Finally, we will use keywords for personalization and real-time functions to limit the results to interventions that have reported these design features. We expect to perform narrative syntheses for each of the 3 target design features. Study quality will be evaluated using the Risk of Bias 2 assessment tool.

**Results:**

We have conducted a preliminary search of existing systematic reviews and review protocols on mHealth-supported behavior change interventions. We have identified several reviews that aimed to evaluate the efficacy of mHealth behavior change interventions in a range of populations, evaluate methodologies for assessing mHealth behavior change randomized trials, and assess the diversity of behavior change techniques and theories in mHealth interventions. However, syntheses on the unique features of mHealth intervention design are absent in the literature.

**Conclusions:**

Our findings will provide a basis for developing best practices for designing mHealth tools for sustainable behavior change.

**Trial Registration:**

PROSPERO CRD42021261078; https://tinyurl.com/m454r65t

**International Registered Report Identifier (IRRID):**

PRR1-10.2196/39093

## Introduction

One of the most significant achievements in human health in the past century was the extension of life expectancy from 45 to >75 years, resulting in an expanding, aging global population [1]. Concurrently, lifestyles have changed with industrialization, inducing dramatic shifts in the global disease burden, which is now dominated by chronic diseases [2,3]. Globally, the leading modifiable risk factors for chronic diseases are poor diet, alcohol consumption, smoking, and a lack of physical activity [4]. More importantly, these lifestyle behaviors are also modifiable factors for chronic disease management [5]. Hence, sustaining healthy lifestyle behaviors has been recommended by the World Cancer Research Fund [6], the International Society of Hypertension [7], the International Diabetes Federation [8], and many others [9-12].

Since the 1950s, the harm of smoking has been embedded in medical training with policy support to promote smoking cessation [13]. Coupled with individual behavior change strategies, smoking rates have seen a continuous decline since the 1960s [14]. In sharp contrast, the inclusion of diet and physical activity as topics in medical training was only initiated as recently as the 21st century [15-18]. Unique to quitting smoking, which removes one behavior, initiating and maintaining healthy dietary patterns and an active lifestyle (ie, reducing sedentary behavior and increasing physical activity) requires sustained behavior changes throughout life. Therefore, responsibility for these behaviors ultimately falls on patients, who must self-manage their chronic diseases in the long term, even with the availability of system and policy supports [19].

Theory-based behavior change interventions are highly efficacious in controlled experiments. Multiple behavior change theories have been tested in selected populations (eg, the Theory of Planned Behavior and the Social Cognitive Theory), targeting individual knowledge and cognitive and affective determinants of behavior. To reduce the complexity of using multiple theories, the Theoretical Domains Framework was developed in 2005 by bringing together 33 models of behavior change [20]. In 2011, the Behaviour Change Wheel was created as a causal “behavior system” to guide intervention design through mapping Theoretical Domains Framework–based behavior determinants to the Behaviour Change Technique Taxonomy [21].

Despite high efficacy in experimental settings, the real-world application of behavior theories has had limited effectiveness at the population level in achieving sustainable changes in diet, physical activity, and sedentary behavior [22,23]. For models that emphasize the interaction between individuals and the environment within a social system (eg, the Ecological Model), environmental influences and policy context often become the primary target [24], risking disparities within population subgroups (ie, by creating urban-rural disparities). Human behavior is dynamic, and sustained behavior change following interventions is dependent on the individual’s ability to adopt and continuously use behavior change techniques [25-27]. Intervention fidelity (ie, the delivery-receipt-enactment chain) is the key process measure of the mechanism linking intervention to outcome [28]. The process of enactment is the most sensitive to potential breakdowns in the delivery-receipt-enactment chain, occurring when individual-level factors and contextual resources are not properly aligned to support enactment [25]. Hence, real-time personalized interventions are desired to improve self-enactment and fidelity by facilitating the continuous alignment of individual and contextual factors.

The growing computing capabilities of mobile phones have enabled us to monitor and deliver health metrics continuously in real time [29,30]. Therefore, mobile health (mHealth) has the potential to enable personalized, real-time feedback and monitoring of targeted behaviors. The term “mHealth” describes the practice of medicine and public health supported by mobile devices [31]. The World Health Organization has recommended mHealth as a key health promotion strategy to improve global health across low- to middle- and high-income countries [32]. In recent years, mHealth has increasingly been used as a method in behavioral research for disease prevention and self-management through supporting positive changes in diet, physical activity, and sedentary behavior [33].

It is important to note that technology-enabled mHealth tools are twofold, including the active ingredients of behavior change interventions as the intervention content and the mHealth tools themselves as the intervention delivery strategy [34]. The computational power (ie, how fast a system can process data and perform a computational task) of mHealth tools can provide unique functions compared to conventional interventions, such as providing personalized behavior change recommendations and delivering them in real time with the support of dialogue systems. It is also worth noting that mHealth interventions differ from in-person interventions in the resources they deliver. For instance, mHealth interventions may deliver behavior change techniques as an end product with virtual resources [35], whereas conventional interventions may be able to deliver direct physical resources, such as in-person group interventions, exercise equipment, or access to facilities [36]. However, design principles for mHealth interventions to incorporate personalization, real-time functions, and deliverable resources have not been systematically evaluated.

The goal of this review is to identify best practices for the design of mHealth interventions targeting diet, physical activity, and sedentary behavior changes for chronic disease prevention and self-management. We aim to review and identify specific designs in current mHealth tools that feature personalization, real-time functions, and deliverable resources. Our findings will provide a basis for developing best practice guidelines for designing mHealth tools targeting sustained behavioral change.

## Methods

### Prospective Registration and Reporting Guidelines

This systematic review has been registered with PROSPERO (CRD42021261078), an international database of prospectively registered systematic reviews. Conduct of this review will be guided by the 2022 updated PRISMA (Preferred Reporting Items for Systematic Reviews and Meta-Analyses) guidelines [37].

### Eligibility Criteria

We will focus on randomized controlled trials that have reported using mHealth tools for interventions targeting diet, physical activity, or sedentary behavior for chronic disease prevention or management purposes among adults aged 18 years or older. The inclusion criteria are as follows: (1) the study must include a behavior change intervention for chronic disease prevention and self-management; (2) the mHealth tool must include a personalization feature (eg, personalized intervention content and dose, delivery process, or feedback) or a real-time feature (eg, real-time behavioral monitoring or a dialogue system); (3) the behavior change need not have been the primary aim of the study or the primary outcome measure of the study, but a specific measure of behavior change must be reported; (4) a deliverable resource does not have to have been included in the mHealth intervention, although we will review and summarize deliverable resources (eg, virtual social support, access to facilities, diet recipes, or exercise videos) in the mHealth tool; (5) the study must be peer-reviewed and must also have an English abstract, even if it is written in a different language. We will screen non-English abstracts for inclusion and leverage international colleagues for full-text screening and data extraction.

### Information Sources

Two reviewers will independently search electronic databases, including MEDLINE, CINAHL, Embase, PsycINFO, and Web of Science, for studies published since 2010 that report findings from mHealth interventions. The year 2010 was chosen because it was when several national and international health organizations identified mHealth as a health promotion strategy with funding opportunities [32,38].

### Search Strategy

We will use keywords for mHealth, behavior change, interventions, and self-management and combine them using the “AND” term (Multimedia Appendix 1). Literature found with the search will discuss mHealth interventions targeting lifestyle behavior changes for chronic disease prevention and self-management. Next, we will use keywords for diet, physical activity, and sedentary behavior to limit the search to mHealth interventions targeting these behaviors. Finally, we will use keywords for personalization and real-time functions, respectively, to limit the identified interventions to ones that have reported these design features.

### Selection Process

The search strategy will be applied to all databases and aggregated in Endnote reference management software (Clarivate LLC). One reviewer will remove obviously irrelevant references by screening the titles and abstracts, with a 5% sample of these decisions being verified by another reviewer. All remaining abstracts will be assessed for inclusion by one reviewer, with all those selected for exclusion being checked by the other reviewers before final exclusion. The full text of all remaining studies will be obtained and assessed independently for inclusion by 2 reviewers, with any discrepancies resolved in discussion with a third reviewer. The process of study selection will be reported in PRISMA flow diagrams [37] and the reasons for exclusion will be noted. Reference lists of the included studies will also be reviewed to further identify relevant studies.

### Data Collection Process

The lead reviewer will develop a data extraction form. Two reviewers will independently pilot the data extraction form on 5 studies. Extracted data will be reviewed by the entire review team to refine the data extraction form. At the commencement of data extraction, 2 reviewers will each extract the data from half of the included studies and perform a cross-check to verify the extracted data. Any discrepancies will be recorded and resolved by discussion.

### Data Items

The following data will be extracted for each included study: first author name, year of publication, journal, country, setting, and objective; study design and content of the mHealth intervention (ie, the targeted behavior, behavior change theory used, personalization features, real-time functions, and deliverable resources); procedures for defining, recruiting, and sampling from the intervention and control groups; characteristics and sample size of the study population; frequency and duration of follow-up; definition and measures of behavior change; reference group in any statistical modeling and results of any statistical tests reported; and subgroup analyses or any evidence relating to effects on other health outcomes.

### Study Risk of Bias Assessment

Study quality will be assessed using the Risk of Bias 2 assessment tool, an update to the original Cochrane risk of bias tool [39]. The Risk of Bias 2 tool evaluates the following domains in randomized controlled trials: randomization process, deviations from the intended interventions, missing outcome data, measurement of the outcome, and selection of the reported result. Two reviewers will assess study quality independently, and their assessments will be compared for agreement, with any discrepancies resolved in discussion with a third reviewer.

### Synthesis Methods

Based on our knowledge and findings from an initial search, we expect substantial heterogeneity in the mHealth interventions across the targeted chronic conditions, targeted behavior (ie, diet, physical activity, and sedentary behavior), reported outcomes, and methods (ie, frameworks and technologies) to achieve personalization, real-time functions, and deliverable resources. Hence, meta-analysis is unlikely to be feasible or appropriate. Therefore, we will perform narrative syntheses following the Reporting Guidelines of Synthesis Without Meta-Analyses [40] guideline for each of the 3 design features of interest.

We will first present detailed descriptions of the included studies in both narrative and tabular formats. This description table will focus on reporting the year of publication, country, setting, study objectives, population, mHealth intervention, comparison group, and outcome measures. Next, the included mHealth interventions will be further evaluated for 3 key design features based on the targeted behavior (ie, diet, physical activity, or sedentary behavior): personalization, real-time functions, and deliverable resources. The study team will develop a separate table for each targeted behavior to map the personalization (ie, personalized intervention content and dose, delivery process, and feedback), real-time functions (ie, the inclusion of technologies to support real-time behavior monitoring and a dialogue system), and deliverable resources (ie, social support, access to facilities, diet recipes, and exercise videos). Finally, the effectiveness of the included mHealth interventions will be presented by showing their unique features, that is, features that they do not share with usual care or nonintervention. A narrative synthesis of the included mHealth interventions will be presented together with an evaluation of study quality (ie, the risk of bias assessment) to provide context for the study findings and support confidence in our evaluation of the state of the field.

Although we anticipate a low likelihood of quantitative synthesis, meta-analyses for outcomes that include a sufficient number of studies will be considered if deemed feasible. We will provide statistical descriptions for behavior change related to the mHealth intervention for specific targeted behaviors and design features, such as personalization and real-time functions. We will estimate the summary effect size and its 95% CI through both fixed and random effects models. Between-study association will be estimated using the *I*² metric; values of 50% are indicative of high heterogeneity, while values above 75% suggest very high heterogeneity [41]. Whenever necessary, we will calculate the evidence of small-study effects (ie, whether small studies have inflated effect sizes compared to larger ones). To this end, we will use the regression asymmetry test developed by Egger and colleagues [42]. A *P* value of .10 with more conservative effects in large studies in random-effects meta-analyses is considered indicative of a small-study effect.

## Results

As of November 10, 2022, we have completed our database search and have begun searching by hand. After removing 607 duplicates, the initial search yielded 2961 studies; the review team will screen the titles, abstracts and full texts. We aim to complete the review by March 2023.

## Discussion

This systematic review will provide a comprehensive overview of the literature to better understand the design of mHealth tools and their unique features, such as support for personalization, real-time functions, and deliverable resources, in interventions targeting diet, physical activity, and sedentary behavior. The main contribution of our review will be an understanding of the current methods and technologies used in mHealth interventions. Any amendments or modifications made to the protocol will be reported in the final paper.

Lifestyles and environments have changed in modern society with industrialization, inducing dramatic shifts in the global disease burden, which is now dominated by chronic diseases [43]. As such, now more than ever, we must face the consequences of the massive societal burden of chronic diseases. Importantly, lifestyle behaviors are both a major cause of chronic diseases and the key to effective management of chronic diseases [44]. Therefore, it is critical to develop tools to support positive changes in lifestyle behaviors and to support individuals in adopting environmental resources for sustainable behavior change. To this end, mHealth tools offer promising avenues to deliver personalized interventions in real time, powered by technology and computing capacity. The coverage rate of mobile technology worldwide increased from 87% to 95% from 2011 to 2012 and is expected to rise to 96% by 2026 [45]. However, best practices for designing mHealth tools to support positive behavior change are unclear.

We have conducted a preliminary search for existing systematic reviews and review protocols on mHealth-supported behavior change interventions. We identified several reviews that aimed to evaluate the efficacy of mHealth behavior change interventions in a range of populations [46-48], evaluate methodologies for assessing mHealth behavior change randomized trials [49], and assess the diversity of behavior change techniques and theories in mHealth interventions [35,48]. However, syntheses of features unique to mHealth intervention design, including personalization, real-time functions, and deliverable resources, are lacking in the literature.

Based on the synthesized data, the key outcomes of our review will be (1) identifying gaps in the existing literature and (2) informing future research to improve the design of mHealth interventions and incorporate their unique features to support sustainable behavior change. These findings will be summarized and reported in a peer-reviewed journal.

## Appendix

Multimedia Appendix 1 Search syntax.

## Abbreviations

mHealth: mobile health
PRISMA: Preferred Reporting Items for Systematic Reviews and Meta-Analyses

## Data Availability

The data sets generated during and/or analyzed during the current study will be made available from the corresponding author on reasonable request.
