# Supplementary material for: Design Principles in mHealth Interventions for Sustainable Health Behavior Changes: Protocol for a Systematic Review
Source: JMIR Res Protoc. 2023 Feb 22;12:e39093. doi: 10.2196/39093 (PMC9996417; doi:10.2196/39093)
Supplement: Multimedia Appendix 1 [file resprot_v12i1e39093_app1.docx]

Multimedia Appendix 1: search strategy

|  |  | MeSH | Keywords |
| --- | --- | --- | --- |
| 1 | mHealth | Exp mobile application/ | (ehealth or e-health or mhealth or m-health or m health or mobile health or web* app* or telehealth or app-based or telemedicine or mobile app* or tele-health or smart-phon* app). or Exp mobile application/ |
| 2 | Behaviour change | Exp health behaviour/ | Behavio* change or behavio* modif* or behavio* or life style* or life-style* or lifestyle* or lifestyle behavio* or behavio* treatment or behavio* theory  or exp health behavior/ |
| 3 | Self management | Exp Self management/  Exp self care/ | self-management or self management or self-care or self care or patient driven or health promotion or illness management or disease management or prevention or Exp Self management/ or exp self care/ |
| 4 | RCT | Exp Randomized Controlled Trial/ | Random* control* trial* or RCT or Exp Randomized Controlled Trial/ |
| 5 | personalization |  | customiz* or tailor* or individual* or personal* |
